# Complete Response to Nivolumab in Recurrent/Metastatic HPV-Positive Head and Neck Squamous Cell Carcinoma Patient After Progressive Multifocal Leukoencephalopathy: A Case Report

**DOI:** 10.3389/fonc.2021.799453

**Published:** 2022-01-10

**Authors:** Laura Deborah Locati, Mara Serena Serafini, Andrea Carenzo, Silvana Canevari, Federica Perrone, Ester Orlandi, Serena Delbue, Stefano Cavalieri, Giulia Berzeri, Anna Pichiecchio, Lisa Francesca Licitra, Enrico Marchioni, Loris De Cecco

**Affiliations:** ^1^ Head and Neck Cancer Medical Oncology Unit, Fondazione Istituto di Ricovero e Cura a Carattere Scientifico (IRCCS) Istituto Nazionale dei Tumori, Milan, Italy; ^2^ Integrated Biology Platform, Department of Applied Research and Technology Development, Fondazione Istituto di Ricovero e Cura a Carattere Scientifico (IRCCS) Istituto Nazionale dei Tumori, Milan, Italy; ^3^ Fondazione Istituto di Ricovero e Cura a Carattere Scientifico (IRCCS) Istituto Nazionale dei Tumori, Milan, Italy; ^4^ Pathology Department, Fondazione Istituto di Ricovero e Cura a Carattere Scientifico (IRCCS) Istituto Nazionale dei Tumori, Milan, Italy; ^5^ Radiation Oncology Clinical Department, National Center of Oncological Hadrontherapy, Pavia, Italy; ^6^ Department of Biomedical, Surgical and Dental Sciences, University of Milan, Milan, Italy; ^7^ Department of Brain and Behavioral Sciences, University of Pavia, Pavia, Italy; ^8^ Advanced Imaging and Radiomics Center, Neuroradiology Department, Istituto di Ricovero e Cura a Carattere Scientifico (IRCCS) Mondino Foundation, Pavia, Italy; ^9^ Department of Hematology and Oncology, University of Milan, Milan, Italy; ^10^ Neuroncology Unit, Istituto di Ricovero e Cura a Carattere Scientifico (IRCCS) Mondino Foundation, Pavia, Italy

**Keywords:** immunotherapy, HNSCC, oropharynx, HPV, case report, PML

## Abstract

In an immune-competent context nivolumab showed long-term benefit in overall survival in recurrent/metastatic head and neck squamous cell carcinoma (HNSCC); however, in special cancer population such as these patients with immunodeficiency and viral infections, data on checkpoint inhibitors (ICI) activity are scant. Herein, we report a patient with a Human papilloma virus (HPV)-related oropharyngeal cancer (OPC) and CD4 lymphocytopenia. After a first-line treatment complete remission, the patient experienced Human Polyomavirus (JCV) infection in the brain. Consequently, to the recovery from progressive multifocal leukoencephalopathy (PML) the patient metastasized and was enrolled in a single-arm trial with nivolumab (EudraCT number: 2017-000562-30). A complete and durable response (more 3 years) was observed after 10 nivolumab injections Q2wks, interrupted for persistent drug related G2 diarrhea and a syndrome of inappropriate antidiuretic hormone secretion. We describe the circulating immune profile (before-, during-, and after nivolumab), consistent with the clinical history. Moreover, during nivolumab treatment, brain MRI evidenced the presence of small punctuate areas of contrast enhancement, reflecting a mild immune response in perivascular spaces. By cytofluorimetry, we observed that during JCV infection the CD4/CD8 ratio of the patient was under the normal values. After JCV infection recovery and before nivolumab treatment, CD4/CD8 ratio reached the normality threshold, even if the CD4^+^ T cell count remained largely under the normal values. During ICI, gene expression xCell analyses of circulating immune cells of the patient, showed a progressive normalization of the total immune profile, with significant boost in CD4^+^ and CD8^+^ T cells and a reduction in NK T, comparable to the circulating immune profile of reference tumor-free HNSCC patients. The present case supports the activity of ICI in a population of special cancer patients; whether JCV and HPV infections (alone or together) might have a possible role as immune booster(s), require further investigations.

## Introduction

Head and neck squamous cell carcinoma (HNSCC) is the eighth most frequent cancer in the world (1). For patients with recurrent/metastatic (R/M) HNSCC the overall survival has been recently improved due to available new treatment options, especially immune checkpoint inhibitors (ICI, i.e., nivolumab, pembrolizumab). Nivolumab is a human immunoglobulin G4 (IgG4) antibody targeting programmed death-1 (PD-1), an inhibitory receptor on activated T cells. This antibody blocks the interaction with PD-1 receptor and its ligands, PD-L1 and PD-L2, leading to the inhibition of the patient immune response against cancer cells. Nivolumab was approved by the FDA for platinum-refractory R/M HNSCC patients and, as reported in the randomized trial CheckMate-141, showed benefit as compared to standard of care and, once response is obtained, it may be prolonged (2). However, despite those encouraging results, in this set of patients response is still limited (about 15%), and to our knowledge predictive biomarkers, such as PD-L1 expression, are still under investigation (3). At our knowledge, data on the activity of nivolumab in challenging populations (such as immunocompromised patients or those with HIV and viral hepatitis infections) are scant, mainly due to their under representation in clinical trials (4, 5). The present unique case report represents a remarkable portrait of peripheral blood immune profile before, during, and post nivolumab treatment in a platinum-refractory R/M HNSCC patient, with two concomitant viral infections, experiencing a long-term response.

## Case Presentation

**Figure 1:**
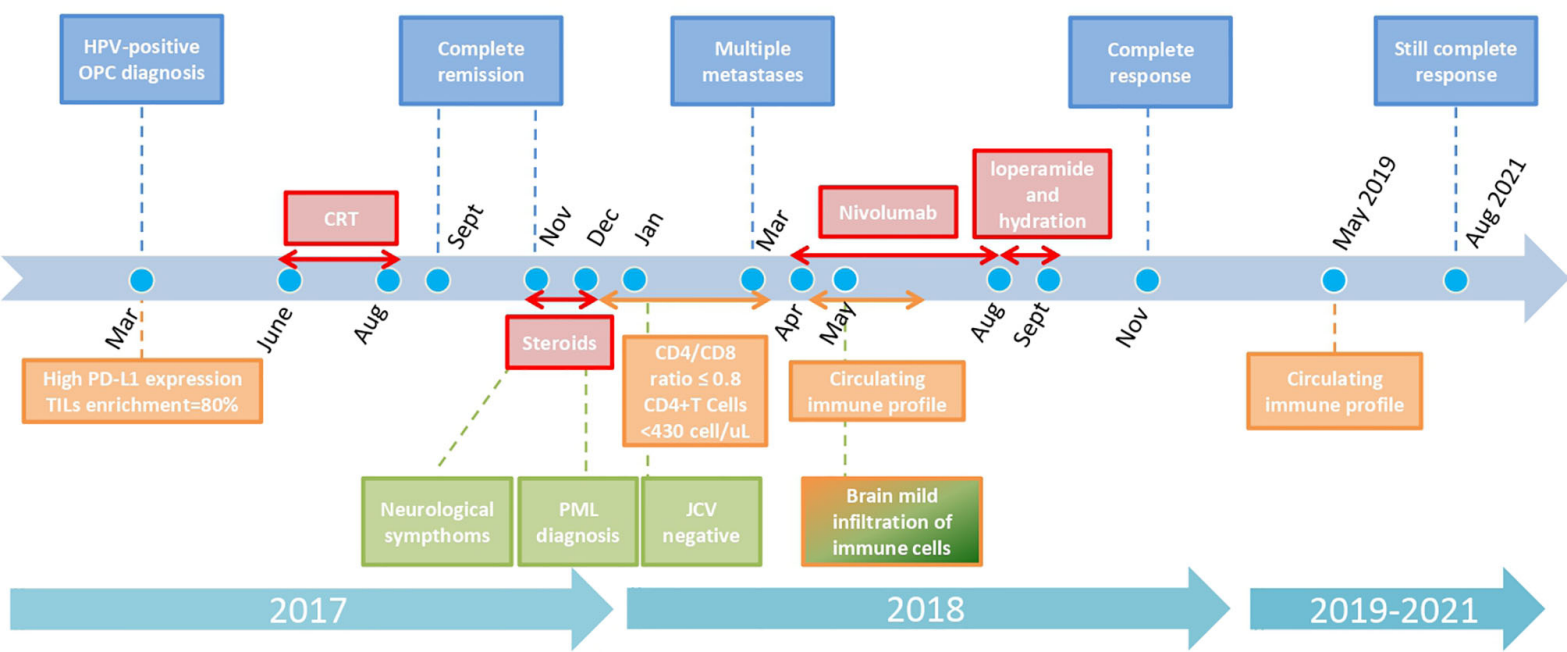
Graphical timeline of case report with key oncologic, neurologic, and immunologic analyses and treatments. Color code for boxes: blue: oncologic history; red: treatments; light green: neurologic history; orange: immunologic analyses results. Every arrow indicates the duration of each treatment/immunologic situation. Dots represent specific key months. The scale is not strictly proportioned to dates.

**Figure 2:**
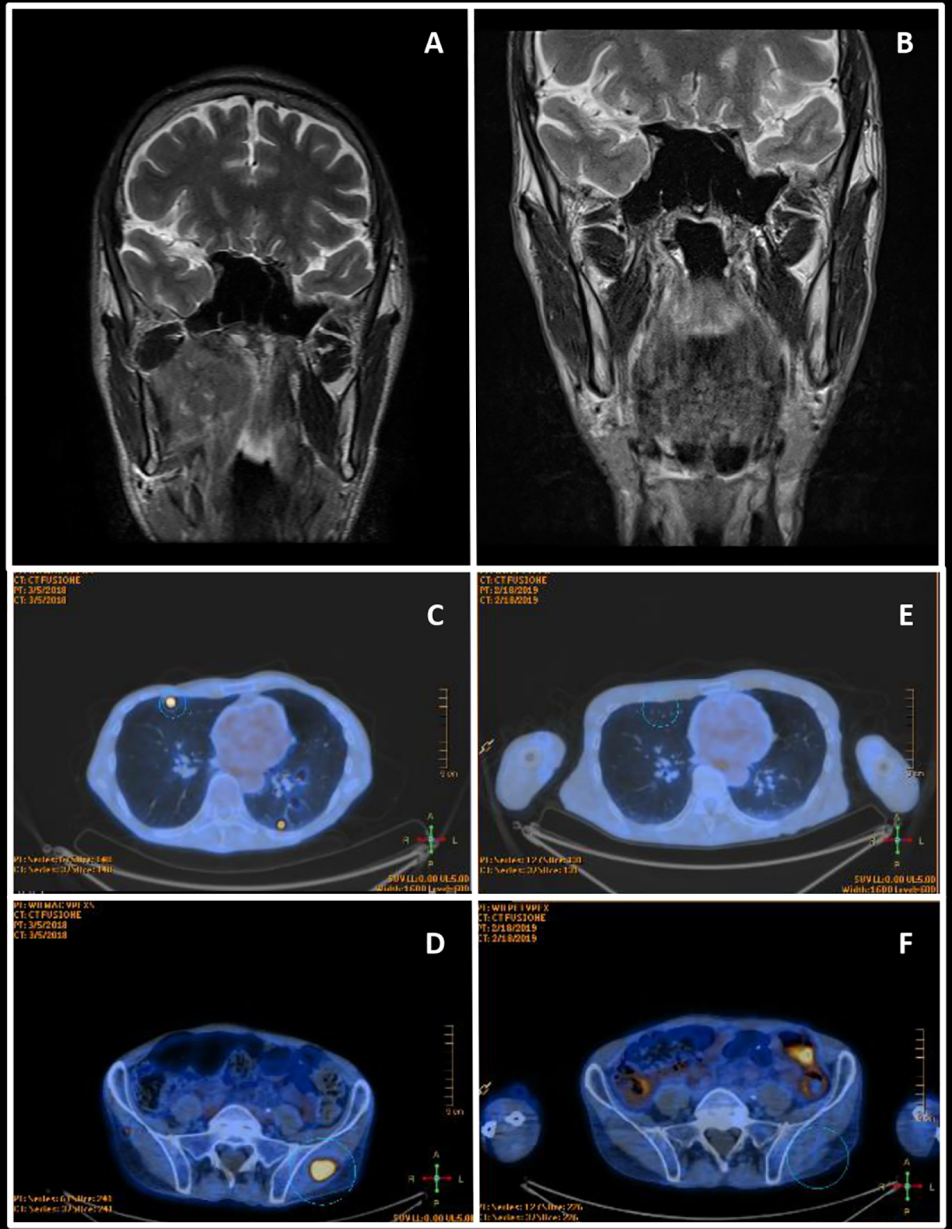
Radiological assessments. **(A)** March 2017 Head and neck contrast-enhanced magnetic resonance imaging—before the primary treatment. **(B)** November 2017 Head and neck contrast-enhanced magnetic resonance imaging of complete remission after chemoradiation. **(C, D)** March 2018 Whole body FDG PET—at diagnosis of metastatic disease. **(E, F)** November 2018 Whole body FDG PET of complete response after nivolumab.

A 62-year-old man, an active smoker (>10 packs/year), was referred to our center (IRCCS Istituto Nazionale dei Tumori, Milan, Italy) in March 2017 due to a large lesion of the right infratemporal fossa (55 × 30 × 52 mm). His medical history was unremarkable apart from previous episodes of paroxysmal atrial fibrillation. A graphical representation of the case history is shown in **Figure 1**. Contrast head and neck magnetic resonance imaging (MRI) revealed a large mass with infiltration of the medial pterigoid muscles and the deep parotid lobe, until the lateral nasopharyngeal wall; posterior, the lesion infiltrated the prevertebral muscles, enclosing the right internal carotid together with multiple enlarged nodes in the ipsilateral neck (**Figure 2A**). Fine needle aspiration biopsy of the largest neck node (25 mm) demonstrated a poorly differentiated carcinoma, p16 immuno-histochemistry (IHC) positive in formalin-fixed, paraffin-embedded (FFPE) sections (6) and HPV viral infection confirmed by *in situ* hybridization (ISH). The patient was classified as cT4 N1 p16 positive, stage III according to the AJCC VIII classification. IHC analysis for PD-L1 expression was performed using IHC 22C3 pharmDx kit (Dako, Carpinteria, CA) on FFPE tissue. PD-L1 expression was evaluated both in tumor cells and in inflammatory cells and a combined proportion score (CPS) was determined and tumor infiltrating lymphocytes (TILs) were assessed on hematoxilin & eosin (H&E) slides (7). The analysis showed high PD-L1 expression both in tumor cells (40%) and infiltrating cells (20%) with a CPS = 55. In addition, we observed an enrichment (80%) of TILs. No residual FFPE tissue was available for additional deepen analysis.

## First-Line Oncologic Treatment and Neurological History

**Figure 3:**
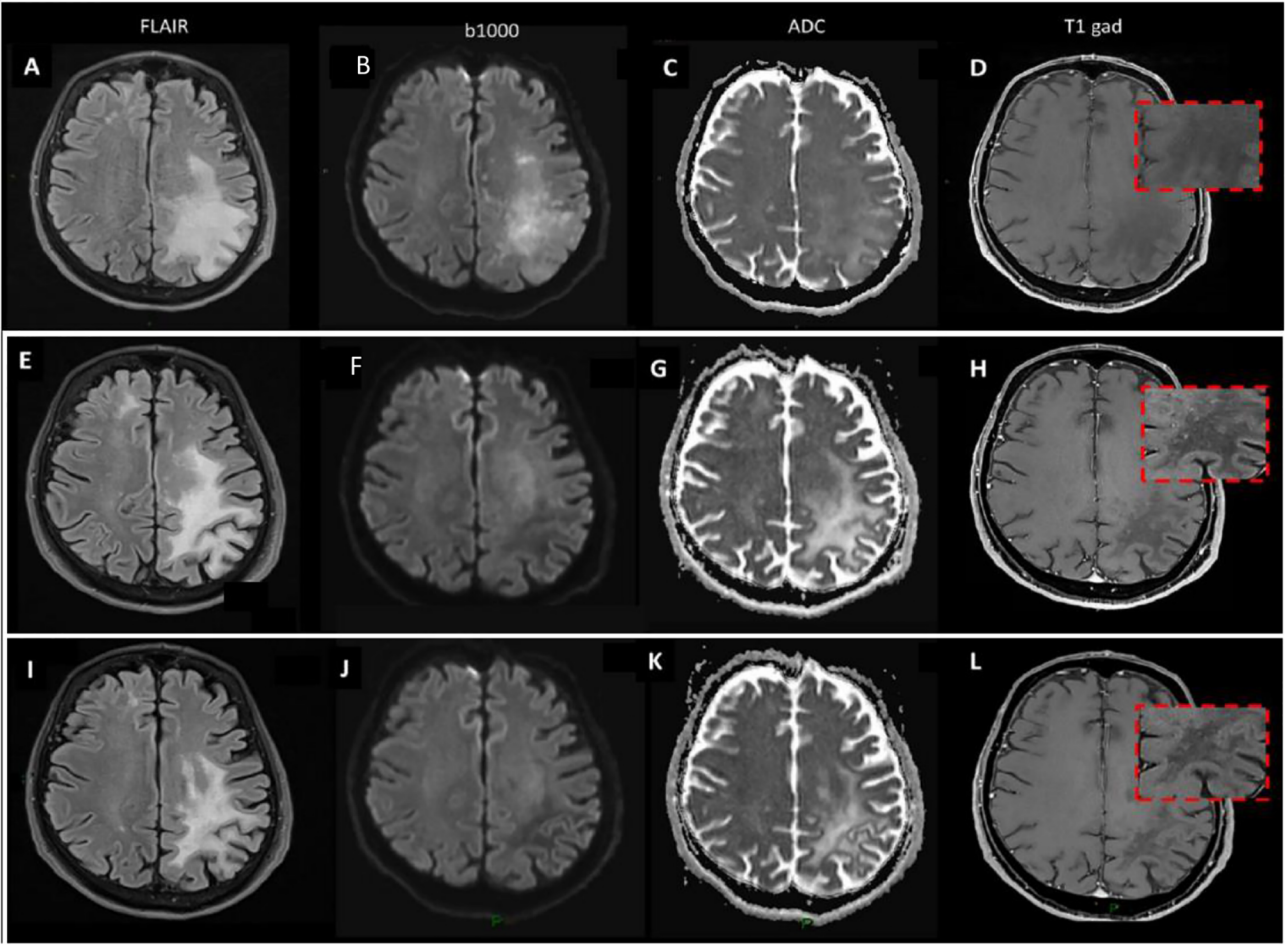
Brain MRI. **(A–D)** December 2017 brain MRI at PML diagnosis. **(E–H)** May 2018 brain MRI during ICI treatment. **(I–L)** January 2019 brain MRI after ICI treatment.

From June to August 2017 the patient received intensity-modulated radiation therapy (IMRT, 69.96 Gy in 33 fractions) concomitant to 2 courses of carboplatin (AUC 6), due to the previous paroxysmal atrial fibrillation, serum creatinine value at the upper level and tumor related weight loss. The patient obtained a complete clinical remission at the end of the treatment, despite an expected CRT myelotoxicity (**Figure 2B**). In November 2017, due to a subacute progressive gait ataxia and aphasia, a contrast brain head and neck MRI was performed (data not shown). The image confirmed the complete oncologic remission and in addition showed multiple T2 hyperintense alterations in the supra- and infratentorial white matter, not correlated with the oncological clinical history. From November till December 2017 the patient was treated with steroids (oral cortisone acetate; 62.5 mg daily), but no improvements of neurological symptoms were observed, and the patient was hospitalized at the Fondazione Istituto Neurologico Mondino (Pavia, Italy). Neurological examination revealed moderate cognitive impairment, severe mixed aphasia, mild right hemiparesis, and cerebellar ataxia. The patient needed support for most daily life activities (modified Rankin scale—mRS—equal to 3) (8). Cerebrospinal fluid (CSF) analysis showed no inflammatory findings and no oligoclonal banding; Human Polyomavirus JC (JCV) DNA was isolated from 150 ml of CSF, using the commercial kit Nuclesopin RNA virus (Macherey Nagel, Germany) and Real Time PCR, targeting the JCV Large- Antigen gene (9). Active JCV replication (4,700 copies/ml) was observed, and a brain MRI performed in December (**Figures 3A–D**), identified a large lesion in the left fronto-parietal white matter, characterized by hyperintensity on T2/FLAIR images, mild mass effect, and no contrast enhancement after gadolinium administration; b1000 images showed small areas of increased signal in a star-like («milky way») distribution pattern, though with incomplete correspondence hyposignal in the ADC map, which is a typical finding in “definite progressive multifocal leukoencephalopathy (PML)”, reflecting diffusion restriction due to active demyelination. Steroid was withdrawn and over the following weeks, the patient experienced a gradual neurological improvement. Since January 2018, JCV viral load became undetectable in the CSF. In March 2018, aphasia and cognitive deficits were substantially improved and he was able to walk independently (mRS 2). Blood tests of lymphoid components, through cytofluorimetric analysis, were performed from December 2017 to March 2018. In December, during steroid treatment, CD4/CD8 ratio was inverted (CD8 >CD4; CD4/CD8 = 0.6, normal threshold >0.8), and the number circulating CD4^+^ T cells were below the normal threshold (between 430 and 1,600 cells/µl) with an impaired count of 73 CD4^+^ cells/µl. In January 2018, with the withdrawal of steroid, a slight improvement in CD4^+^ T cell count was observed (161 cells/µl) and CD4/CD8 ratio was restored (CD4 >CD8, CD4/CD8 = 1.4). In March 2018, CD4/CD8 ratio was in the normal threshold (CD4/CD8 = 08), and CD4^+^ T cell count was slightly increasing 165 cell/µl. However, CD4^+^ T cells remained largely under the normal threshold.

## Multiple Distant Metastasis and Second-Line Treatment

In March 2018, multiple distant metastases in skin, soft tissues, and bilateral lungs have been found at the follow-up 18FDG-PET scan (**Figures 2C, D**). In April 2018, the patient was enrolled in the study “A Single-Arm, Open-Label, Multicenter, Trial with Nivolumab in Subjects with Recurrent or Metastatic Platinum-refractory Squamous Cell Carcinoma of the Head and Neck” (EudraCT number: 2017-000562-30). During the following six months, the patient received 10 well-tolerated nivolumab infusions, each one of 240 mg every two weeks. A brain MRI, performed after the 4th nivolumab infusion (**Figures 3E–H**), showed a reduction in the extension of the left fronto-parietal lesion that no longer displayed mass effect, as evident on FLAIR images; b1000 images now showed an extensive area of hyposignal with correspondent hypersignal on ADC, reflecting extensive white matter destructuration without signs of ongoing active demyelination; areas of slight increased b1000 signal were still evident more anteriorly. T1 images after gadolinium injection showed the appearance of small punctuate areas of contrast enhancement, reflecting a mild immune response in perivascular spaces. Only a mild aphasia and apraxia without further deficits were present and JC viral load in the CSF continued to be undetectable. Blood tests, through cytofluorimetric analysis, at the 5th cycle of nivolumab detected a CD4/CD8 ratio in the normal threshold (CD4/CD8 = 08), while CD4^+^ T cell count remained largely under the normal values (160 cells/µl). In June 2018 a partial response according to the RECIST 1.1 criteria (5) was evident at the CT scan. In September 2018, nivolumab was withdrawn after 10 infusions, due to a G2 diarrhea (probably drug-related), persistent despite symptomatic treatment, and a syndrome of inappropriate antidiuretic hormone secretion (SIADH). Diagnosis of immune-mediated colitis was not endoscopically confirmed. Side effects were managed with loperamide and hydration, without using steroid or immunosuppressive therapy because of the risk of JC reactivation. In January 2019 a contrast brain MRI (**Figures 3I–L**) confirmed a substantial stability of imaging findings, disappearing of diffusivity abnormalities, but further evolution of cortical atrophy, even more evident at later follow-up evaluations. T1 images after gadolinium showed the disappearance of the small punctuate areas of enhancement that were previously evident during nivolumab treatment. In November 2019, a complete remission was confirmed by a CT scan total body (**Figures 2E, F**). At date, October 2021, the patient is still in complete remission for the HPV-related OPC and the JC infection.

## Circulating Immune-Profile Results

Recognizing the importance of profiling the immunological characteristics of the patient, blood samples collected before, during and after the single agent nivolumab treatment were employed to investigate, through a de-convolution gene expression method, the immune cells populations present in the peripheral blood. As reference, blood samples were also collected from 5 different HNSCC patients tumor-free after radical treatment. The reference patients were in the same age range and of the same gender (but one) of our patient, while they are heterogeneous in smoking history (3 heavy ex-smokers, 2 never smokers), site of the primary disease (two were p16 positive OPCs, 3 oral cavity cancer). Blood collection was made after signed informed consent from each patient. To perform the analysis, RNA was extracted from frozen blood buffycoat using RNeasy Lipid Tissue Mini Kit (Qiagen). Quality/quantity RNA had been assessed by 2200TapeStation system (Agilent), and Qubit 2.0 Fluorimetric Assay (Thermo Fisher Scientific), respectively. Gene expression experiments were performed using GeneChip WT Pico standard protocols (Affymetrix, ThermoFisher) on human Clariom-S chips. Probes were hybridized on GeneChip, that after washing and staining, through the Fluidics Station, were scanned with Affymetrix Gene Chip Scanner 3000 7G. Microarray data were compliant to MIAME (Minimum Information about a Microarray Experiment) and two gene expression matrices (5 different temporal samples from case report; 5 samples from reference patients) were deposited on GEO (accession number GSE161785). Bioinformatic analysis were performed on two different expression matrices, by the xCell approach (10).

**Figure 4:**
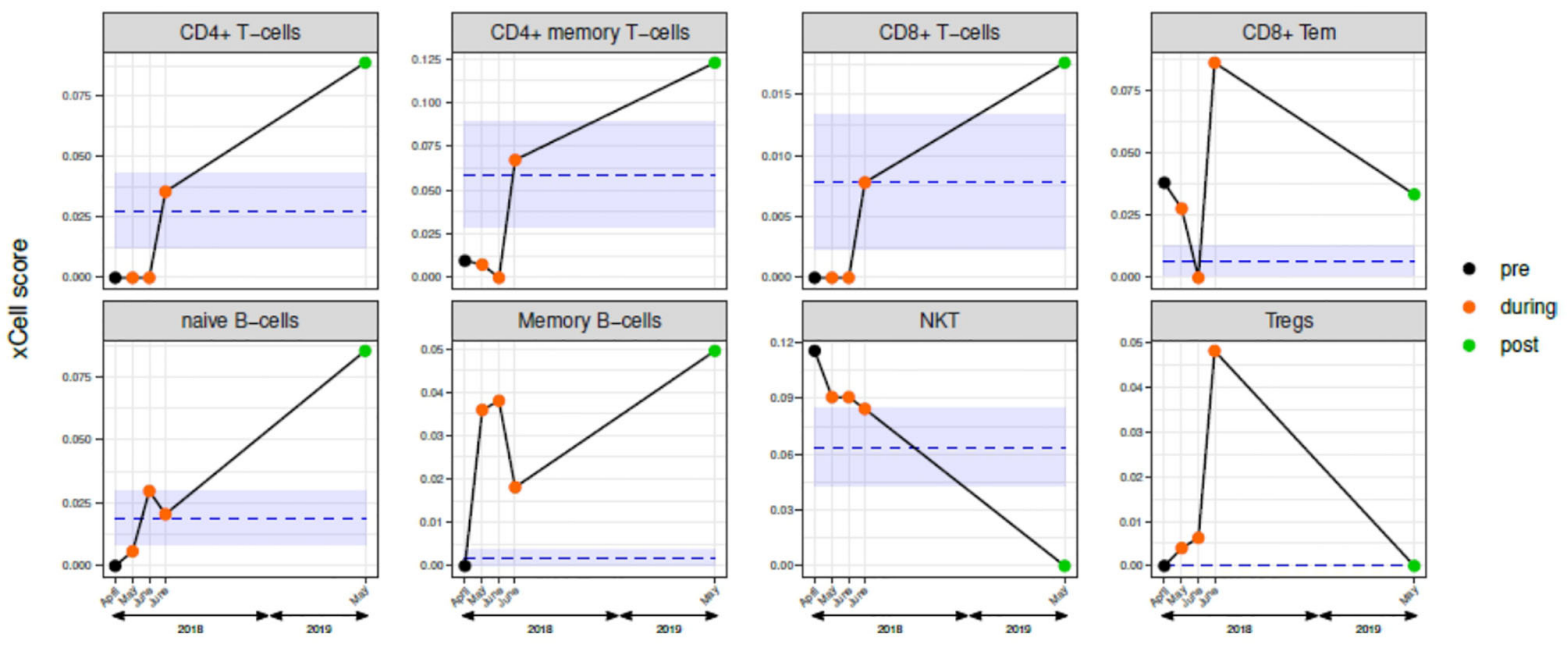
Circulating immune cells characterization before, during, and after nivolumab treatment. Scores of selected lymphoid cells determined by xCell analysis of gene expression data of blood samples from: five samples case report patient (one pre-nivolumab: black dot; three during-nivolumab: orange dots; one post-nivolumab: green dot); mean and standard deviation of the five-reference xCell scores of tumor-free patients are represented by blue lines and light blue area, respectively. See **Table S1** for xCell data for the complete xCell scores (“lymphocytes” and “non-lymphocytes”).

Lymphoid cells with evident changes during and after nivolumab are reported in **Figure 4** (see **Supplementary Table** for all the xCell results and related statistics). At the beginning of April 2018 (“before-nivolumab”), all the considered lymphoid cells, with exclusion of NKT and CD8^+^ Tem, exhibited a lower or absent expression compared to controls. In May and June 2018 in two different blood samples (“during-nivolumab”), no relevant differences were observed for CD4^+^ T cells, CD4^+^ memory T cells, CD8^+^ T cells, naïve B cells, and Tregs, compared to the baseline expression. On the contrary, a decrease for CD8^+^ Tem and NKT and an increase for memory B cells were recorded. In comparison with the references’ range of expression, the major immunological profile changes were recorded in the 3rd sample (7th nivolumab infusion): CD4^+^ T cells, CD4^+^ memory T cells, CD8^+^ T cells, naïve B cells, NKT entered in the range, while CD8^+^ Tem, memory B cells, and Tregs overtook the reference range. Interestingly, the immunological boost observed during the last sample “during-nivolumab”, overtaking the controls, has been maintained one year later in May 2019.

## Discussion

We describe here the complete clinical and radiological remission of a metastatic, platinum-refractory, HPV-related OPC with ICI favorable markers (tumor PD-L1 and TILs IHC data) (3) in a patient who, after a partial recovery from CRT myelotoxicity and a complete recovery from a JC viral infection, received nivolumab. To our knowledge this is the first report of a complete durable response after ICI in a cancer patient with two viral infections (HPV in the tumor and JC in the cerebral system). Immunotherapy has been clinically used for the treatment of PML in patients with hematological malignancies, HIV-infection, or primary immune deficiencies, with erratic and conflicting results. In fact, while most patients show a decrease of PD1 expression on T-CD4^+^ and CD8^+^ lymphocytes as a result of PD1 blockade, only a proportion of them shows a consensual improvement of PML and even negative effects in transplant-receiver patients (11). In our case, PML was challenging and lately diagnosed since the patient had no personal history suggesting a primary immune deficiency and nivolumab was administrated after a transient immune suppression and PML remission. In the PML patients treated with ICI (12, 13), T cell profile seems the major determinant in eliciting a clinical and radiological response, being patients with higher amounts of terminally exhausted T cells less likely to respond to PD1 blockade (11, 14). Similar observations have been reported in patients receiving ICI for cancer treatment (15) and specifically in HNSCC (16). For these reasons, we decided to analyze the circulating immune profile of the case report patient before, during and after nivolumab, using the gene expression of frozen buffy coat and, as reference, HNSCC patients tumor-free after first-line treatment. The choice was based on the available material according to the trial protocol and on the evidence that gene expression data are comparable to cytofluorimetric data (10). At baseline we observed an immunoprofile consistent with the clinical history of our patient, with low levels of effector T cells (CD4^+^ and CD8^+^), and higher levels of CD8^+^ memory and NKT-cells, in contrast with those reported for HNSCC patients (13), where, in an immunocompetent context, the response to nivolumab at baseline was associated with higher levels of CD8^+^ T cells and lower levels of PD-1^+^ Tregs. Even if the baseline immune profile of our patient could be interpreted as a negative predictor of response, the significant increase in relative levels of total CD4^+^ T and memory B cells during ICI, maintained after the end of nivolumab, was in agreement with ICI efficacy. In addition, if we consider the particular immune status of this patient and we compare our data with those of PML patients, the increase of CD4^+^ T cells during treatment seems consistent with the observation that CD4^+^ T cell counts increased during ICI in responding PML patients (15). Indeed, the brain MRI during ICI showed the appearance of small punctuate areas of contrast enhancement within PML lesions, not present at the time of PML diagnosis, which spontaneously resolved, after nivolumab discontinuation. These areas, similarly to literature reported cases of ICI treated PML (17), are described as immune cell infiltrates in perivascular spaces and might be due to a collateral nivolumab boosting of a JC-specific cellular immunity. Nonetheless, PML was already evolving favorably in our patient before the start of nivolumab, questioning the contribution of nivolumab in JC virus clearance. Overall, the immune profile changes during treatment and the clinical outcome suggested that the immunotherapy was effective despite a persistent CD4^+^ lymphocytopenia. Although the immune system impairment, we decided to no deepening into the lymphopenia of our patient since it might be specifically attributed to chemo-radiation treatment and steoroid administration, frequently recorded in HNSCC patients after primary treatment (18).

We should acknowledge some limitations of our study. First, the lack amount of available tumor tissue precluded the comparison between circulating immune cells with tumor immune microenvironment. Second, in adherence to the study protocol, the use of the entire blood samples for RNA extraction precluded a deeper evaluation of the intriguing Tregs expression during treatment, and long-term maintenance of high levels of memory B cells in comparison with samples of references. Third, the comparison with the literature reports (17) is indirect because an absolute counts of immune cells by cytofluorimetry has been reported in the literature, while our immune-profile was inferred from gene expression data. In conclusion, nivolumab induced a durable and complete response in a metastatic HNSCC patient with CD4 lymphocytopenia, experiencing two consecutive viral infections such as HPV and JCV. Our findings support the use of ICI in immune compromised subjects but whether JCV alone or in association with HPV infection could act as immune booster(s) requires further investigations.

## Data Availability Statement

The datasets presented in this study can be found in GEO repository (ID GSE161785). The names of the repository/repositories and accession number(s) can be found below: https://www.ncbi.nlm.nih.gov/geo/query/acc.cgi?acc=GSE161785.

## Ethics Statement

Ethical review and approval was not required for the study on human participants in accordance with the local legislation and institutional requirements. The patients/participants provided their written informed consent to participate in this study. Written informed consent was obtained from the individual(s) for the publication of any potentially identifiable images or data included in this article.

## Author Contributions

Conception and design: LDL, LDC, and SiC. Financial support: LDC. Provision of study material: LDL and FP. Collection and assembly of data: LDL, EO, SD, GB, AP, and EM. Experimental data generation: MSS and LDC. Data analysis and interpretation: MSS, LDC, AC, SiC, StC, EM, and LFL. Manuscript writing: SiC, LDL, MSS, StC, LDC, and EM. All authors contributed to the article and approved the submitted version.

## Funding

The research leading to these results has received funding from AIRC (IG23573 project—P.I. LDC).

## Conflict of Interest

LFL Receipt of grants/research supports (Funds received by my institution for clinical studies and research activities in which I am involved): Astrazeneca, BMS, Boehringer Ingelheim, Celgene International, Debiopharm International SA, Eisai, Exelixis inc, Hoffmann-La Roche ltd, IRX Therapeutics inc, Medpace inc, Merck–Serono, MSD, Novartis, Pfizer, Roche. Receipt of honoraria or consultation fees (for public speaking/teaching in medical meetings and/or for expert opinion in advisory boards): Astrazeneca, Bayer, BMS, Eisai, MSD, Merck–Serono, Boehringer Ingelheim, Novartis, Roche, Debiopharm International SA, Sobi, Ipsen, Incyte Biosciences Italy srl, Doxa Pharma, Amgen, Nanobiotics Sa and GSK. LDL Receipt of grants/research supports (Funds received by my institution for clinical studies and research activities in which I am involved): EISAI Receipt of honoraria or consultation fees (for public speaking/teaching in medical meetings and/or for expert opinion in advisory boards): EISAI, IPSEN, BMS, MSD, Merck Serono, McCann Healthcare, Sanofi, SunPharma, Eli Lilly. LDC Receipt of grants/research supports (Funds received by my institution for clinical studies and research activities in which I am involved): AIRC.

The remaining authors declare that the research was conducted in the absence of any commercial or financial relationships that could be construed as a potential conflict of interest.

## Publisher’s Note

All claims expressed in this article are solely those of the authors and do not necessarily represent those of their affiliated organizations, or those of the publisher, the editors and the reviewers. Any product that may be evaluated in this article, or claim that may be made by its manufacturer, is not guaranteed or endorsed by the publisher.
