# Supplementary material for: Complete Response to Nivolumab in Recurrent/Metastatic HPV-Positive Head and Neck Squamous Cell Carcinoma Patient After Progressive Multifocal Leukoencephalopathy: A Case Report
Source: Front Oncol. 2022 Jan 10;11:799453. doi: 10.3389/fonc.2021.799453 (PMC8784387; doi:10.3389/fonc.2021.799453)
Supplement: Supplementary file 1 [file Table_1.docx]

**SUPPLEMENTS**

**Supplementary Tables**

**Table S1.1 xCell scores of the five case report’s blood samples**

| **ID** | **pre** | **during 1** | **during 2** | **during 3** | **post** | **Correlation to Time (Spearman)** | **one-sided significance** | **Group** |
| --- | --- | --- | --- | --- | --- | --- | --- | --- |
| **Days to baseline** | **0** | **29** | **57** | **84** | **418** |  |  |  |
|  |  |  |  |  |  |  |  |  |
| aDC | 0 | 0.0095 | 0.0024 | 0.0012 | 0 | -0.205196 | 0.370291 | Non-lymphocytes |
| Basophils | 0 | 0.0426 | 0 | 0 | 0 | -0.353553 | 0.2797022 | Non-lymphocytes |
| B-cells | 0 | 0.04 | 0.0435 | 0.0289 | 0.1109 | 0.7 | 0.0940602 | Lymphocytes |
| CD4+ memory T-cells | 0.0097 | 0.0075 | 0 | 0.0673 | 0.1229 | 0.6 | 0.1423785 | Lymphocytes |
| CD4+ naive T-cells | 0 | 0 | 0 | 0 | 0.0168 | 0.7071068 | 0.0908451 | Lymphocytes |
| CD4+ T-cells | 0 | 0 | 0 | 0.0355 | 0.0887 | 0.8944272 | 0.0202597 | Lymphocytes |
| CD4+ Tcm | 0 | 0 | 0 | 0 | 0 | 0 |  | Lymphocytes |
| CD4+ Tem | 0 | 0.0169 | 0 | 0.0101 | 0.0342 | 0.615587 | 0.1344989 | Lymphocytes |
| CD8+ naive T-cells | 0.0264 | 0 | 0 | 0.0317 | 0 | -0.111803 | 0.4289723 | Lymphocytes |
| CD8+ T-cells | 0 | 0 | 0 | 0.0078 | 0.0176 | 0.8944272 | 0.0202597 | Lymphocytes |
| CD8+ Tcm | 0 | 0 | 0 | 0 | 0.0177 | 0.7071068 | 0.0908451 | Lymphocytes |
| CD8+ Tem | 0.0382 | 0.0278 | 0 | 0.0865 | 0.0335 | 0.1 | 0.4364443 | Lymphocytes |
| cDC | 0.0453 | 0.0153 | 0.035 | 0 | 0.0188 | -0.5 | 0.1955011 | Non-lymphocytes |
| Class-switched Memory B-cells | 0 | 0.0457 | 0.0568 | 0.0355 | 0.0633 | 0.7 | 0.0940602 | Lymphocytes |
| DC | 0.0089 | 0 | 0.0267 | 0 | 0 | -0.447214 | 0.2250924 | Non-lymphocytes |
| Eosinophils | 0 | 0 | 0.0359 | 0 | 0 | 0 | 0.5 | Non-lymphocytes |
| iDC | 0.0116 | 0 | 0.0167 | 0 | 0 | -0.447214 | 0.2250924 | Non-lymphocytes |
| Macrophages | 0 | 0 | 0 | 0 | 0.0017 | 0.7071068 | 0.0908451 | Non-lymphocytes |
| Macrophages M1 | 0 | 0 | 0.0006 | 0.0027 | 0 | 0.3354102 | 0.2905453 | Non-lymphocytes |
| Macrophages M2 | 0.0012 | 0 | 0.0043 | 0.0019 | 0.004 | 0.6 | 0.1423785 | Non-lymphocytes |
| Mast cells | 0 | 0 | 0 | 0.0045 | 0.0008 | 0.7826238 | 0.0588069 | Non-lymphocytes |
| Memory B-cells | 0 | 0.0359 | 0.038 | 0.0181 | 0.0495 | 0.7 | 0.0940602 | Lymphocytes |
| Monocytes | 0.0445 | 0.0068 | 0.0221 | 0 | 0 | -0.872082 | 0.0269271 | Non-lymphocytes |
| naive B-cells | 0 | 0.0058 | 0.0297 | 0.0205 | 0.0854 | 0.9 | 0.018693 | Lymphocytes |
| Neutrophils | 0.0189 | 0.0476 | 0.0874 | 0 | 0.03 | -0.1 | 0.4364443 | Non-lymphocytes |
| NK cells | 0.0693 | 0.0218 | 0 | 0.1412 | 0 | -0.307794 | 0.307192 | Lymphocytes |
| NKT | 0.1156 | 0.0907 | 0.0907 | 0.0844 | 0 | -0.974679 | 0.0024091 | Lymphocytes |
| pDC | 0 | 0.0146 | 0 | 0 | 0.0213 | 0.4472136 | 0.2250924 | Non-lymphocytes |
| Plasma cells | 0 | 0.0055 | 0 | 0 | 0.0033 | 0.1118034 | 0.4289723 | Lymphocytes |
| Platelets | 0.0139 | 0.0325 | 0 | 0.0645 | 0.0256 | 0.3 | 0.3119188 | Non-lymphocytes |
| pro B-cells | 0 | 0.0211 | 0.0099 | 0 | 0 | -0.33541 | 0.2905453 | Lymphocytes |
| Tgd cells | 0.0201 | 0.0159 | 0 | 0.0184 | 0.0431 | 0.3 | 0.3119188 | Lymphocytes |
| Th1 cells | 0 | 0.029 | 0 | 0 | 0 | -0.353553 | 0.2797022 | Lymphocytes |
| Th2 cells | 0 | 0 | 0 | 0.0259 | 0.0211 | 0.7826238 | 0.0588069 | Lymphocytes |
| Tregs | 0 | 0.004 | 0.0063 | 0.0483 | 0 | 0.2051957 | 0.370291 | Lymphocytes |

**Table S1.2 xCell scores of the five reference patients’ blood samples**

| **ID** | **ref 1** | **ref 2** | **ref 3** | **ref 4** | **ref 5** | **mean** | **SD** | **Group** |
| --- | --- | --- | --- | --- | --- | --- | --- | --- |
| aDC | 0 | 0 | 0 | 0.0018 | 0 | 3.60E-04 | 8.05E-04 | Non-lymphocytes |
| Basophils | 0.0431 | 0.0491 | 0 | 0 | 0.0812 | 0.03468 | 0.03481504 | Non-lymphocytes |
| B-cells | 0 | 0 | 0.0439 | 0.0455 | 0 | 0.01788 | 0.024489733 | Lymphocytes |
| CD4+ memory T-cells | 0.0077 | 0.0358 | 0.0685 | 0.1833 | 0 | 0.05906 | 0.074484314 | Lymphocytes |
| CD4+ naive T-cells | 0 | 0.0144 | 0 | 0 | 0 | 0.00288 | 0.006439876 | Lymphocytes |
| CD4+ T-cells | 0 | 0.0184 | 0.0272 | 0.0925 | 0 | 0.02762 | 0.038145537 | Lymphocytes |
| CD4+ Tcm | 0 | 0 | 0 | 0 | 0.0097 | 0.00194 | 0.004337972 | Lymphocytes |
| CD4+ Tem | 0 | 0 | 0 | 0.0847 | 0 | 0.01694 | 0.037878992 | Lymphocytes |
| CD8+ naive T-cells | 0.0104 | 0 | 0.0317 | 0.0628 | 0.0371 | 0.0284 | 0.024497449 | Lymphocytes |
| CD8+ T-cells | 0 | 0.0318 | 0 | 0.0077 | 0 | 0.0079 | 0.013770258 | Lymphocytes |
| CD8+ Tcm | 0 | 0.0028 | 0 | 0.0411 | 0 | 0.00878 | 0.018108065 | Lymphocytes |
| CD8+ Tem | 0 | 0.033 | 0 | 0 | 0 | 0.0066 | 0.014758049 | Lymphocytes |
| cDC | 0 | 0 | 0.0427 | 0.0488 | 0.0062 | 0.01954 | 0.024156324 | Non-lymphocytes |
| Class-switched memory B-cells | 0 | 0.0497 | 0.0266 | 0 | 0.0143 | 0.01812 | 0.020859458 | Lymphocytes |
| DC | 0 | 0 | 0 | 0 | 0.0036 | 7.20E-04 | 0.001609969 | Non-lymphocytes |
| Eosinophils | 0.0207 | 0.0632 | 0 | 0.017 | 0.1844 | 0.05706 | 0.074893044 | Non-lymphocytes |
| iDC | 0.0212 | 0.0443 | 4.00E-04 | 0 | 0.0435 | 0.02188 | 0.021855594 | Non-lymphocytes |
| Macrophages | 0.0139 | 0.007 | 0.0398 | 0.0245 | 0 | 0.01704 | 0.015610029 | Non-lymphocytes |
| Macrophages M1 | 0 | 0 | 0 | 0 | 0 | 0 | 0 | Non-lymphocytes |
| Macrophages M2 | 0.0265 | 0.0493 | 0.0808 | 0 | 0.0175 | 0.03482 | 0.031246872 | Non-lymphocytes |
| Mast cells | 0.0195 | 0.0148 | 0.0165 | 0.0075 | 0 | 0.01166 | 0.007874198 | Non-lymphocytes |
| Memory B-cells | 0 | 0 | 0.0096 | 0 | 0 | 0.00192 | 0.004293251 | Lymphocytes |
| Monocytes | 0.0599 | 0.0069 | 0.0277 | 0 | 0 | 0.0189 | 0.025577627 | Non-lymphocytes |
| naive B-cells | 0 | 0 | 0.0378 | 0.0563 | 0 | 0.01882 | 0.026587441 | Lymphocytes |
| Neutrophils | 0.0218 | 0.0573 | 0.0386 | 0 | 0.041 | 0.03174 | 0.021750586 | Non-lymphocytes |
| NK cells | 0 | 0 | 0.0469 | 0.0285 | 0 | 0.01508 | 0.021649642 | Lymphocytes |
| NKT | 0 | 0.0259 | 0.0641 | 0.1293 | 0.0998 | 0.06382 | 0.052662102 | Lymphocytes |
| pDC | 0 | 0 | 0 | 0.0201 | 0 | 0.00402 | 0.008988993 | Non-lymphocytes |
| Plasma cells | 0 | 0 | 9.00E-04 | 0 | 0 | 1.80E-04 | 4.02E-04 | Lymphocytes |
| Platelets | 0.1004 | 0.1199 | 0.0323 | 0.054 | 0 | 0.06132 | 0.049027411 | Non-lymphocytes |
| pro B-cells | 0 | 1.00E-04 | 0 | 0 | 0.035 | 0.00702 | 0.015641355 | Lymphocytes |
| Tgd cells | 0 | 0.0073 | 0 | 0.0146 | 0 | 0.00438 | 0.006529318 | Lymphocytes |
| Th1 cells | 0.0406 | 0 | 0.0694 | 0.0309 | 0.045 | 0.03718 | 0.025163307 | Lymphocytes |
| Th2 cells | 0 | 0 | 0 | 0.0418 | 0.0114 | 0.01064 | 0.018104916 | Lymphocytes |
| Tregs | 0 | 0 | 0 | 0 | 0 | 0 | 0 | Lymphocytes |
